# A Comparative Study of Leptospirosis and Dengue in Thai Children

**DOI:** 10.1371/journal.pntd.0000111

**Published:** 2007-12-26

**Authors:** Daniel H. Libraty, Khin S. A. Myint, Clinton K. Murray, Robert V. Gibbons, Mammen P. Mammen, Timothy P. Endy, Wenjun Li, David W. Vaughn, Ananda Nisalak, Siripen Kalayanarooj, Duane R. Hospenthal, Sharone Green, Alan L. Rothman, Francis A. Ennis

**Affiliations:** 1 Center for Infectious Disease and Vaccine Research, University of Massachusetts Medical School, Worcester, Massachusetts, United States of America; 2 Department of Virology, US Army Medical Component, Armed Forces Research Institute for Medical Sciences, Bangkok, Thailand; 3 Brooke Army Medical Center, Ft. Sam Houston, Texas, United States of America; 4 Department of Preventive and Behavioral Medicine, University of Massachusetts Medical School, Worcester, Massachusetts, United States of America; 5 Department of Pediatrics, Queen Sirikit National Institute of Child Health, Bangkok, Thailand; Institut Pasteur, France

## Abstract

**Background:**

Leptospirosis is an emerging zoonosis that is often under-recognized in children and commonly confused with dengue in tropical settings. An enhanced ability to distinguish leptospirosis from dengue in children would guide clinicians and public health personnel in the appropriate use of limited healthcare resources.

**Methodology/Principal Findings:**

We conducted a prospective, hospital-based, study of children with acute febrile illnesses and dengue in Thailand. Among the children without dengue, we identified those with leptospirosis using anti-leptospira IgM and microscopic agglutination titers in paired acute and convalescent blood samples. We then performed a case-control comparison of symptoms, signs, and clinical laboratory values between children with leptospirosis and dengue.

In a semi-rural region of Thailand, leptospirosis accounted for 19% of the non-dengue acute febrile illnesses among children presenting during the rainy season. None of the children with leptospirosis were correctly diagnosed at the time of hospital discharge, and one third (33%) were erroneously diagnosed as dengue or scrub typhus. A predictive model to distinguish pediatric leptospirosis from dengue was generated using three variables: the absolute neutrophil count, plasma albumin, and aspartate aminotransferase levels in the first 72 hours of illness.

**Conclusions/Significance:**

Unrecognized leptospirosis can be a significant cause of “dengue-like” febrile illness in children. Increased awareness of pediatric leptospirosis, and an enhanced ability to discriminate between leptospirosis and dengue early in illness, will help guide the appropriate use of healthcare resources in often resource-limited settings.

## Author Summary

Two of the most common causes of acute febrile illnesses among children in the tropics are leptospirosis and dengue. Early in illness, these two conditions are often indistinguishable and rapid laboratory confirmation of the infecting pathogen is generally not available. An enhanced ability to distinguish leptospirosis from dengue in children would guide clinicians and public health personnel in the appropriate use of limited healthcare resources.

In a prospective, hospital-based, study of children with acute febrile illnesses conducted in Thailand, we compared clinical and laboratory characteristics between children with leptospirosis and dengue. Unrecognized leptospirosis was a significant cause of “dengue-like” febrile illness among children presenting to a semi-rural hospital during the rainy season. The presenting symptoms and physical examination findings were poor at discriminating between children with leptospirosis and dengue. A predictive model to distinguish pediatric leptospirosis from dengue was generated using three laboratory values on hospital admission. Our results provide important information on the characteristics of pediatric leptospirosis in Southeast Asia and its distinguishing features from dengue in a region where both pathogens circulate.

## Introduction

Leptospirosis is an increasingly recognized cause of acute febrile illness throughout the tropical and sub-tropical regions of the world. Spirochetal infection with *Leptospira sp.* typically occurs when water or soil contaminated with the urine of an infected animal comes in contact with human skin or mucous membranes [1],[2]. The clinical manifestations of leptospirosis range from a mild self-limited febrile illness to a severe and potentially fatal illness characterized by jaundice, renal failure, thrombocytopenia, and hemorrhage (Weil's disease). Early in illness, leptospirosis is often indistinguishable from other common causes of acute febrile illnesses in the tropics- e.g. dengue, malaria, scrub typhus, typhoid, and others [1],[3]. Children in particular often bear the brunt of these tropical diseases, and pose the greatest diagnostic challenges to clinicians. In the pediatric population, leptospirosis and dengue often have similar clinical manifestations and are among the most common diagnostic dilemmas. Both typically occur during the rainy season, and rapid laboratory confirmation of the infecting pathogen is generally not available. Several studies have shown that leptospirosis is often confused with dengue and under-diagnosed in endemic regions [3]–[5].

We conducted a prospective study of acute febrile illnesses and suspected dengue virus (DENV) infections among children presenting to hospitals at two sites in Thailand. Children were diagnosed with an acute DENV infection based on virological and serological criteria (see methods). Those without DENV infection, and no evidence of bacterial infection or malaria, were classified as having other febrile illnesses (OFIs). Among the children with OFIs, we were able to test acute and convalescent blood samples for evidence of an acute leptospiral infection. Our results provide important information on the characteristics of pediatric leptospirosis in Southeast Asia and its distinguishing features from dengue in a region where both pathogens circulate.

## Methods

### Study design and population

Details of the investigational protocol have been published previously [6]. Children included in this study were seen at the Queen Sirikit Institute of Child Health in Bangkok, Thailand between 1994–1999, and at the Kamphaeng Phet Provincial Hospital, Kamphaeng Phet, Thailand between 1994–1997. The Queen Sirikit Institute of Child Health is a tertiary-level medical center in a large metropolitan area. The Kamphaeng Phet Provincial Hospital is a secondary-level facility in a semi-rural province 360 kilometers north of Bangkok. The investigational protocol was approved by the Institutional Review Boards of the Thai Ministry of Public Health, the Office of the U.S. Army Surgeon General, and the University of Massachusetts Medical School. Parents or guardians of all study subjects gave written informed consent.

Enrollment criteria were age 6 months–14 years, a febrile illness with <72 hours of symptoms, no hypotension or shock, and no other obvious source of infection. Children were observed in hospital until at least 1 day after defervescence. All physical exam findings were recorded by one of five study physicians and experienced pediatricians. Venous blood samples were drawn daily from admission up to the day after defervescence or for a maximum of 5 consecutive days. An early convalescent blood sample was also obtained 8–13 days after enrollment. All clinical data were abstracted onto standardized case report forms. Serial daily tourniquet tests were performed in standardized fashion, as previously described [6]. Day 1 was defined as the calendar day of hospital presentation and study enrollment. A complete blood count (T540 counter, Coulter, Hialeah, FL), and plasma aspartate aminotransferase (AST), alanine aminotransferase (ALT), and albumin determinations (Clinical System Analyzer, model 700, Beckman Instruments, Brea, CA) were obtained daily. Aliquots of plasma were stored at -70°C.

### Initial study classification criteria

DENV infections were identified by a serotype specific RT-PCR assay on study day 1 plasma samples [7], or using previously established serologic criteria for IgM/IgG ELISAs in paired acute and convalescent samples [8]. Children with DENV infections were further classified into dengue fever (DF) and dengue hemorrhagic fever (DHF) grades, according to WHO criteria [9]. Acute febrile illnesses without evidence of DENV infection, routine bacterial infection, or malaria, were classified as OFIs. Leptospirosis diagnostic testing was performed on OFI samples.

### Leptospirosis diagnostic testing and classification criteria

Paired acute and early convalescent plasma samples collected from OFI subjects were screened for anti-leptospira antibody using the PanBio IgM ELISA (PanBio Inc., Brisbane, Australia). Values <9 PanBio ELISA units were considered negative, 9–11 equivocal, and >11 positive, as per the manufacturer's instructions. Subjects who demonstrated a rise in IgM from negative to positive or equivocal values, and those with sustained positive or equivocal values (at both the acute and early convalescent time points), were selected for additional confirmatory testing.

Confirmatory microscopic agglutination testing (MAT) of acute and convalescent plasma samples was performed using a battery of 24 serovars from 20 serogroups selected specifically for use in Southeast Asia. The MAT was conducted in standard fashion, as previously described [10]. Serovars included in the antigen panel were *L. biflexa* serovar Andamana, *L. interrogans* serovar Australis, *L. interrogans* serovar Bratislava, *L. santarosai* serovar Borincana, *L. interrogans* serovar Autumnalis, *L. kirschneri* serovar Butembo, *L. borgpetersenii* serovar Ballum, *L. interrogans* serovar Bataviae, *L. interrogans* serovar Canicola, *L. weilii* serovar Celledoni, *L. interrogans* serovar Hebdomadis, *L.kirschneri* serovar Cynopteri, *L. interrogans* serovar Grippotyphosa, *L. interrogans* serovar Copenhageni, *L. interrogans* serovar Icterohaemorrhagiae, *L. borgpetersenii* serovar Javanica, *L. santarosai* serovar Georgia, *L. interrogans* serovar Pomona, *L. interrogans* serovar Pyrogenes, *L. santarosai* serovar Alexi, *L. borgpetersenii* serovar Tarassovi, *L. interrogans* serovar Hardjo, *Leptospira santarosai* serovar Shermani, *L. borgpetersenii* serovar Sejroe.

Cases with ≥1∶800 MAT antibody titer in a single specimen [11], or a ≥4-fold increase in MAT antibody titers between paired acute and convalescent samples, were classified as definite leptospirosis. Those with MAT antibody titers ≥1∶200 late in illness or at early convalescence, but not meeting the above criteria, were classified as probable leptospirosis. MAT antibody titers ≤1∶100 were considered negative for acute infection. MATs are not adequate for determining the infecting *Leptospire* serovar but can allude to serogroup.

### Statistical analysis

We used the Wilcoxon signed-rank and Mann-Whitney U tests for comparisons of continuous variables not normally distributed. χ^2^ analysis was used for comparisons among proportional data. Multiple logistic regression models were used to assess the association of leptospirosis vs. dengue with relevant clinical and demographic factors. p<0.05 was considered significant. All analyses were carried out using Stata SE 9.1 (StataCorp, College Station, TX).

## Results

### Study population

**Figure 1:**
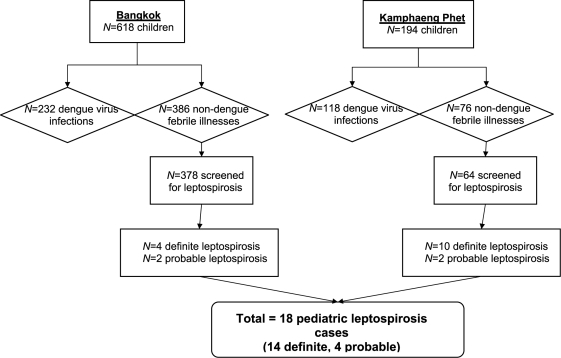
Schematic flow diagram of study subject categorization and leptospirosis testing.

We enrolled 812 children in a prospective study of acute febrile illnesses and suspected DENV infection between 1994–1999 at two sites in Thailand (Bangkok and Kamphaeng Phet). There were 350 children with DENV infections (*n* = 232 in Bangkok, *n* = 118 in Kamphaeng Phet) and 462 children with non-dengue OFIs (*n* = 386 in Bangkok, *n* = 76 in Kamphaeng Phet). We tested for leptospirosis in *n* = 442 (96%) of the children with non-dengue OFIs. At the semi-rural Kamphaeng Phet site, leptospirosis accounted for 12 of the 64 non-dengue acute febrile illnesses that were screened (prevalence 19%). At the metropolitan Bangkok site, the prevalence of leptospirosis within the non-dengue febrile illnesses was only 1.6%- 6 out of 378 cases screened (Figure 1).

Eighteen children were diagnosed with leptospirosis (14 definite, 4 probable). Their median age was 10 years (range 4–14 years), and the male:female ratio was 3.5∶1. All the children with leptospirosis had self-limited illnesses. 7/18 (39%) received antibiotics at some point during their illness, and no serious short-term sequelae of leptospiral infection were noted. One child with leptospirosis developed primary varicella infection immediately after discharge from the hospital. A summary of the leptospirosis cases is presented in Table 1. None of the clinical discharge diagnoses included leptospirosis. One third (33%) of the leptospirosis cases were erroneously diagnosed as scrub typhus (*n* = 2, due to false positive Weil-Felix serology) or dengue (*n* = 6).

**Table 1:** Summary of the pediatric leptospirosis cases.

Case No.^a^	Age (y)	Gender	Day	IgM ELISA^b^ (units)	MAT highest titer^c^ (serovar)	Leptospirosis Diagnosis	Clinical diagnosis at discharge	Comments
1 (KPP)	7	F	2	33.7	1∶3200 (Autumnalis)	Definite	Pharyngitis	
			9	27.9	1∶3200			
2 (KPP)	6	M	1	2.7	neg	Definite	Viral infection	Developed conjunctivitis OD
			8	29.2	1∶3200 (Hebdomadis and Hardjo)			
3 (KPP)	5	M	1	2.5	neg	Definite	Viral infection	
			5	39.6	1∶1600 (Autumnalis)			
4 (KPP)	11	F	1	2.0	neg	Definite	Scrub Typhus	Weil-Felix serology, Proteus OX-K titer 1∶160
			8	49.1	1∶3200 (Andamana)			
5 (KPP)	14	M	1	2.7	neg	Definite	Exudative Tonsillitis	
			9	26.1	>1∶12,800 (Autumnalis)			
6 (KPP)	12	M	1	1.9	1∶400	Definite	Viral infection	Presented with maculopapular rash
			10	20.9	>1∶12,800 (Autumnalis)			
7 (KPP)	11	M	2	35.2	1∶6400	Definite	Gastroenteritis	Developed chickenpox 1 day after discharge
			9	59.1	1∶3200 (Andamana and Canicola)			
8 (KPP)	6	M	1	26.2	neg	Definite	Dengue Fever	
			9	18.7	1∶400 (Cynopteri)			
9 (KPP)	10	M	1	4.1	Neg	Definite	Viral infection	
			9	24.7	1∶12,800 (Autumnalis and Icterohaemorrhagiae			
10 (KPP)	10	F	6	8.2	1∶200 (Autumnalis and Icterohaemorrhagiae)	Probable	Dengue Fever	
			14	11.0				
11 (KPP)	11	M	1	4.1	neg	Definite	Viral infection	Widal test for Salmonella typhi O (1∶40)
			9	40.9	>1∶12,800 (Sejroe)			
12 (KPP)	11	M	1	3.8	1∶400 (Autumnalis)	Probable	Scrub Typhus	Weil-Felix serology, Proteus OX-K titer 1∶80;
			5	14.9				
13 (BKK)	8	M	1	22.4		Definite	Viral infection	
			3	41.3	1∶1600 (Andamana)			
			8	49.2				
14 (BKK)	13	M	1	18.6	1∶200 (Australis)	Probable	Dengue Fever	
			9	16.3	1∶200 (Andamana)			
15 (BKK)	11	M	1	1.3	Neg	Definite	Dengue Hemorrhagic Fever	Subconjunctival hemorrhage noted
			9	63.0	1∶6400 (Canicola)			
16 (BKK)	7	M	2	8.9	neg	Probable	Viral infection	Developed herpes simplex labialis
			9	11.1	1∶200 (Bratislava)			
17 (BKK)	6	F	1	12.8	1∶800	Definite	Viral infection	
			9	10.1	1∶800 (Autumnalis and Cynopteri)			
18 (BKK)	4	M	1	3.6	Neg	Definite	Viral Infection	

a KPP =  Kamphaeng Phet, BKK =  Bangkok.

b PanBio Leptospira IgM ELISA.

c MAT =  leptospira microscopic agglutination titer. Positive results are reported by serovar(s) reactive at the highest titer (lowest dilution). MATs are not adequate for determining the infecting *Leptospire* serovar but can allude to serogroup.

### Epidemiologic characteristics of leptospirosis compared to dengue in a pediatric population

**Figure 2:**
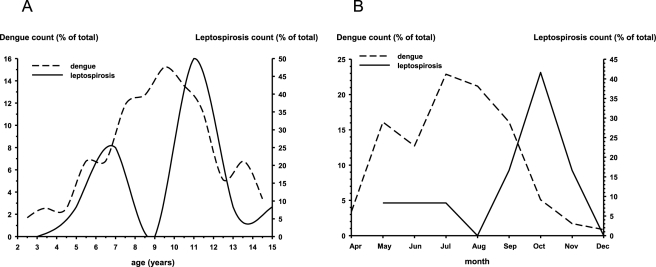
Ages and months of presentation for children with leptospirosis or dengue in Kamphaeng Phet, Thailand. (A) Age distribution of children presenting with leptospirosis (*n* = 12) or dengue (*n* = 118) in Kamphaeng Phet, Thailand. (B) Month of presentation of children with leptospirosis (*n* = 12) or dengue (*n* = 118) in Kamphaeng Phet, Thailand.

During the rainy season in Kamphaeng Phet, Thailand, dengue accounted for 118/194 (61%) of the acute febrile illnesses that prompted study entry. Leptospirosis accounted for 12/194 (6%) of overall acute febrile illnesses, and at least 16% of the non-dengue acute febrile illnesses. We therefore compared some of the epidemiological characteristics between children with dengue and leptospirosis in Kamphaeng Phet (dengue-*n* = 118; leptospirosis-*n* = 12). The age distribution of children presenting with dengue in Kamphaeng Phet was essentially normal with a median age of 9.4 years. The median age of the children with leptospirosis in Kamphaeng Phet was 11.0 years, and the distribution was bimodal with a second small peak around age 6.5 years (Figure 2A). The male∶female ratio was 3∶1 for the leptospirosis cases and 1.3∶1 for the dengue cases, but this difference was not significant (p = 0.12, χ^2^ test). There was also no difference in the number of people in the household, or the number of siblings ill in the household, between children with leptospirosis and dengue (data not shown). The peak occurrence of leptospirosis was in October, 2–3 months later than the peak occurrence of dengue (Figure 2B). Three quarters (75%) of the children with leptospirosis in Kamphaeng Phet presented after August, whereas only 31% of dengue cases did so (p = 0.001, χ^2^ test). Overall, children with leptospirosis in an endemic region presented later in the rainy season, and were slightly older or younger, than their counterparts with dengue.

### Clinical and laboratory characteristics distinguishing leptospirosis vs. dengue in a pediatric population

We next compared clinical and laboratory variables obtained in the children diagnosed with leptospirosis (*n* = 18) to all age-and sex-matched children with DENV infections among the 812 children in the study population from both sites (*n* = 214). The presenting symptoms and signs in the leptospirosis and dengue groups are shown in Table 2. Both groups had a similar prevalence of non-specific and constitutional symptoms early in illness. A minority (≤13%) of the children with leptospirosis or dengue presented with hemorrhage or rash. A greater proportion of children with leptospirosis had <10 petechiae on the admission tourniquet test compared to those with dengue (67% vs. 36%, respectively, p = 0.03, χ^2^ test). By receiver operating characteristic (ROC) analysis, ≤6 petechiae on the admission tourniquet test distinguished leptospirosis from dengue with sensitivity = 61% and specificity = 73%, but only provided a 16% positive predictive value (PPV). On initial physical examination, a palpable liver edge may have been less frequent in the children with leptospirosis compared to those with dengue, but the difference did not reach statistical significance (9% vs. 38%, respectively, p = 0.06).

**Table 2:** Case-control comparison of presenting symptoms and signs between children with leptospirosis and dengue.

Symptom/Sign on presentation	Leptospirosis (*n* = 18)	Dengue (*n* = 214)	p-value^a^
Age (y)	9.5±3.0^b^	8.6±2.5^b^	—
Gender (M∶F)	3.5∶1	2.1∶1	—
No. days of illness before presentation	2.9±0.7^b^	3.1±0.8^b^	NS
Fever (temp≥38.0°C)	100%	100%	—
Anorexia	94%	86%	NS
Headache	72%	84%	NS
Lethargy	78%	87%	NS
Nausea	72%	77%	NS
Vomiting	61%	76%	NS
Abdominal pain	33%	36%	NS
Hemorrhage/bleeding	11%	13%	NS
Rash	6%	1%	NS
Tourniquet Test (no. of petechiae/inch^2^)	(*n* = 18)	(*n* = 208)	**0.03**
<10	67%	36%	
10–19	11%	25%	
≥20	22%	40%	
Palpable liver edge below right costal margin	9%	38%	0.06

a NS =  not significant.

b values are mean±S.D.

The white blood cell (WBC) count and differential on presentation were markedly different in the children with leptospirosis compared to dengue (Table 3). The mean WBC count was 10,531±3,445/mm^3^ with a relative neutrophil predominance (83±11%) and absolute neutrophilia (8,932±3,570/mm^3^) in the children with leptospirosis. The mean WBC count was 5,492±2,807/mm^3^ in those with dengue. The percentage of lymphocytes was higher in dengue than leptospirosis (21±14% vs. 13±9%, respectively, p = 0.01), but an absolute lymphopenia remained (958±670/mm^3^). There were few circulating immature neutrophils (band forms) on admission in those with leptospirosis (<5% in all cases). The percentage of atypical lymphocytes was greater in the children with dengue compared to leptospirosis (3.7±4.4% vs. 1.9±2.9%, dengue vs. leptospirosis, p = 0.01). By ROC analysis, a WBC count ≥6,450/mm^3^ on admission distinguished leptospirosis from dengue with 94% sensitivity and 76% specificity. The mean platelet count on admission trended lower in the children with dengue compared to leptospirosis, but did not achieve statistical significance (Table 3). There were no differences in the admission hemoglobin or hematocrit levels. Mean plasma AST levels were slightly higher in children with dengue compared to leptospirosis. There were no significant differences in ALT levels on admission (Table 3). Mean plasma albumin levels on admission were slightly but statistically significantly lower in the leptospirosis group compared to dengue (4.5±0.5 gm/dl vs. 4.8±0.6 gm/dl, respectively, p = 0.03),

The age-and sex-matched cohort of children with DENV infections ultimately had DF (*n* = 117), DHF Grade I/II (*n* = 82), and DHF Grade III (*n* = 15). Over the course of their acute illness and hospitalization, a greater proportion of the children with dengue developed a petechial rash compared to those with leptospirosis (data not shown). The platelet nadir was lower in children with dengue compared to leptospirosis (99,822±68,949/mm^3^ vs. 197,167±80,477/mm^3^, dengue vs. leptospirosis, p<0.001). Finally, the degree of hepatomegaly that developed during illness was not significantly different between the two groups (maximum liver size detected [cm below right costal margin]-leptospirosis: 0.5 cm [0.3–3.4] vs. dengue 2.0 cm [1.6–2.0], median [95% CI], p = 0.3).

**Table 3:** Case-control comparison of initial laboratory values between children with leptospirosis and dengue. Values are mean±S.D.

Admission Laboratory Value	Leptospirosis (*n* = 18)	Dengue (*n* = 214)	p-value^a^
White blood cell count (cells/mm^3^)	10,531±3,445	5,492±2,807	**<0.001**
% neutrophils	83±11	70±15	**<0.001**
Absolute neutrophil count (cells/mm^3^)	8,932±3,570	4,039±2,660	**<0.001**
% lymphocytes	13±9	21±14	**0.01**
Absolute lymphocyte count (cells/mm^3^)	1,163±687	958±670	NS
% atypical lymphocytes	1.9±2.9	3.7±4.4	**0.01**
Platelets (x10^3^/mm^3^)	245±97	202±83	0.06
hemoglobin (g/dl)	13±0.9	12±1.1	NS
hematocrit (%)	38±3	38±3	NS
AST^b^ (IU/ml)	42±16	66±57	**0.01**
ALT^c^ (IU/ml)	26±14	32±32	NS
Albumin (g/dl)	4.5±0.5	4.8±0.6	**0.03**

a NS =  not significant.

b AST =  aspartate aminotransferase.

c ALT =  alanine aminotransferase.

### A predictive model to distinguish leptospirosis from dengue in children

**Figure 3:**
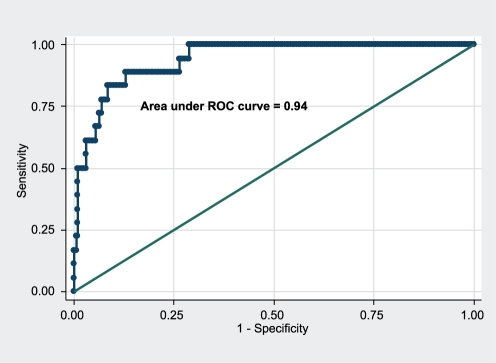
Receiver operating characteristic (ROC) curve of the multivariate logistic regression model for leptospirosis vs. dengue (Table 4). Diagonal line represents the null hypothesis.

Despite statistically significant differences, many individual clinical or laboratory variables were poor discriminators between children with leptospirosis and dengue early in illness. We therefore included the following data obtained on hospital presentation in a logistic regression model: number of petechiae on the tourniquet test, WBC count, absolute neutrophil count (ANC), atypical lymphocyte percentage, platelet count, AST, and albumin levels. A higher probability of leptospirosis compared to dengue was independently associated with higher ANC, lower albumin levels, and AST levels between 30–80 IU/ml on presentation (Table 4). The area under the ROC curve for the predictive model was 0.94 (Figure 3). With a probability cutoff >10%, the predictive model gave a sensitivity of 83%, specificity 90%, PPV 42%, and negative predictive value (NPV) 98% for leptospirosis compared to dengue. A probability cutoff >30% produced a sensitivity of 61%, specificity 96%, PPV 55%, and NPV 96%.

**Table 4:** Multivariate logistic regression model for leptospirosis vs. dengue^a^.

Variable^b^	Unit of change	Multivariate OR [95% CI]^c^	p-value
Absolute neutrophil count	1,000/mm^3^ increase	2.0 [1.5–2.6]	<0.001
Plasma albumin level	1.0 g/dl increase	0.1 [0.03–0.4]	0.001
Plasma AST level^d^	30–80 IU/ml vs. <30 or >80 IU/ml	10.6 [1.8–64.0]	0.01
Platelet count	<200,000/mm^3^ vs. ≥200,000/mm^3^	4.0 [0.9–17.4]	0.06

a All variables entered into model presented in text.

b All variables are admission values obtained within 72 hours of illness onset.

c Multivariate odds ratio (OR) with 95% confidence intervals (CI) for leptospirosis compared to dengue.

d AST =  aspartate aminotransferase.

## Discussion

Leptospirosis in children is often under-diagnosed, especially in those who do not present with the severe icteric form of disease. In a semi-rural region of north central Thailand, we found that leptospirosis accounted for at least 6% of all acute undifferentiated febrile illnesses, and 19% of the non-dengue illnesses, among children presenting to hospital during the rainy season. As expected, the prevalence of leptospirosis was much lower in the metropolitan environment of Bangkok. We used the PanBio IgM ELISA to screen for leptospirosis in well-timed acute and convalescent blood samples, and performed MAT on any equivocal or positive samples. It is unlikely that a large number of leptospirosis cases were missed, since the sensitivity of the PanBio IgM ELISA as used in this study has been reported as high (76–90%) [12],[13]. Some characteristics of the children we identified with leptospirosis were similar to what has been previously reported. There was a male predominance, cases peaked during times of flooding (i.e. late rainy season), and illness was generally less severe than what is typically reported in adults [14]–[17]. Our cohort of children with leptospirosis had self-limited and even milder disease than what has been reported from studies of pediatric leptospirosis in Brazil, Reunion Island, and India [14]–[16],[18],[19]. One reason may be differences in the predominant circulating *Leptospira* serovars. Serovars that have been reported to be associated with severe illness in children are Icterhemorrhagiae, Copenhageni, Canicola, and Sejroe [14],[16]. In our cohort of Thai children with leptospirosis, we noted predominant seroreactivity to serovars Autumnalis and Andamana (serogroups Autumnalis and Andamana, respectively). However, others have reported different serovar associations with disease severity [1] or none at all [20]. Another reason for the mild disease seen here could be the slightly younger age distribution of the children in our study. An age-dependent association with the severity and case-fatality rate of leptospirosis has been observed across many regions of the world [15],[21]. Host factors that may contribute to this association could include higher organism loads with increasing age, or age-dependent changes in innate and adaptive immune responses to leptospiral infection. Finally, 39% of the children received antibiotics, but not because leptospirosis was suspected. We cannot determine if early antibiotic therapy played a role in ameliorating the severity of some pediatric leptospiral disease.

In our cohort of 18 children with confirmed leptospirosis, none were diagnosed correctly by the time of hospital discharge. The most common specific alternative diagnoses were dengue and scrub typhus. Leptospirosis is often indistinguishable from dengue at the critical early stages of illness, and the two are confused commonly [3],[4]. A lack of affordable and accurate diagnostic tests in many settings also contributes to the diagnostic confusion. Predictive models using more readily available clinical and laboratory characteristics would provide useful information to practitioners. We found that presenting symptoms within the first 3 days of illness were not helpful in distinguishing children with leptospirosis from dengue. A lower petechial count on the standardized tourniquet test was associated with leptospirosis compared to dengue in our study and among adult patients in Bangladesh [3]. However, the predictive value of this single test was poor, and the association did not remain significant in a multivariate predictive model. Conjunctival abnormalities were seen in 2/18 (11%) of the children with leptospirosis in this study. Conjunctival inflammation or hemorrhage may have been confused with conjunctival suffusion (with or without hemorrhage), a condition classically described in leptospirosis [22]. The children with leptospirosis also tended to have a lower degree of hepatomegaly than the age-and sex-matched dengue patients. Similar observations were reported among children with leptospirosis and dengue in Mumbai, India [19]. Unfortunately, none of the aforementioned physical signs had sufficient prevalence or discriminatory capability to be useful early in illness.

We found that the most striking difference on presentation between children with leptospirosis and dengue existed in their WBC count and differential. The most significant single laboratory value independently associated with leptospirosis compared to dengue was the absolute neutrophil count (ANC). Neutrophilia is often reported in leptospirosis [22]. However, the ANC has not been previously reported as a useful indicator of leptospiral infection in children [14],[16],[18], or distinguishing leptospirosis from dengue in any age group [3],[19]. The predominance of mature neutrophils and paucity of band forms on the WBC differential might also be an additional useful clue to the diagnosis of leptospirosis. We found that the combination of 3 laboratory values early in illness-ANC, albumin, and AST-provided the best ability to distinguish between leptospirosis and dengue among children with acute undifferentiated febrile illnesses presenting to the hospital. This predictive model will need to be tested and validated in a prospective fashion in order to determine its potential clinical utility.

Our study was limited to symptomatic children presenting to a hospital for evaluation. Its conclusions cannot be extrapolated to the spectrum of disease that does not present to the hospital and may have different clinical and laboratory manifestations. Some potentially useful clinical variables may also not have emerged in our analysis due to the relatively small number of children with leptospirosis. With increased recognition of children with leptospirosis in Kamphaeng Phet, Thailand [23], future comparative studies will have greater statistical and discriminatory power. In the tropics, leptospirosis can be a significant cause of “dengue-like” febrile illness among children presenting to the hospital during the rainy season. Increased awareness of pediatric leptospirosis, and an enhanced ability to discriminate between leptospirosis and dengue early in illness, will help guide the appropriate use of healthcare resources in often resource-limited settings.
